# Morphometric and Molecular Muscle Remodeling after Passive Stretching in Elderly Female Rats

**DOI:** 10.6061/clinics/2020/e1769

**Published:** 2020-11-10

**Authors:** Hilana Rickli Fiuza Martins, Talita G. Gnoato Zotz, Sabrina Peviani Messa, Luiz Guilherme A. Capriglione, Rafael Zotz, Lucia Noronha, Marina Louise Viola De Azevedo, Anna Raquel Silveira Gomes

**Affiliations:** I Programa de Pos Graduacao em Educacao Fisica da Universidade Federal do Parana (PPGEDF-UFPR), Curitiba-Parana. Departamento de Fisioterapia da Universidade Estadual do Centro-Oeste (UNICENTRO) e Centro Universitario GuairacaPrograma de Pos Graduacao em Educacao Fisica da Universidade Federal do Parana (PPGEDF-UFPR)Curitiba-Parana. Departamento de Fisioterapia da Universidade Estadual do Centro-Oeste (UNICENTRO) e Centro Universitario Guairaca, Guarapuava-Parana; II Departamento de Prevencao e Reabilitacao em Fisitoterapia, Universidade Federal do Parana (UFPR) Curitiba, PR, BR; III Faculdade de Ciencias Biomedicas de Cacoal, RO, BR; IV Programa de Ciencias da Saude, Universidade do Parana (PUCPR), Curitiba, PR, BR; V Bacharel em zootecnia, Pontificia Universidade Catolica do Parana (PUCPR), Curitiba, PR, BR; VI Laboratorio da Escola de Medicina e Patologia Experimental, Programa de Pos-Graduacao em Ciencias da Saude, Pontificia Universidade Catolica do Parana (PUCPR) after Programa de Pos Graduacao em Ciencias da Saude; VII Departamento de Patologia Experimental, Pontificia Universidade Catolica do Parana (PUCPR), Curitiba, PR, BR; VIII Departamento de Prevencao e Reabilitacao em Fisitoterapia, Programa de Pos-Graduacao em Educacao Fisica, Universidade Federal do Parana (UFPR) Curitiba, PR, BR

**Keywords:** Muscle-Stretching Exercise, Musculoskeletal System, Extracellular Matrix, Rats, Aging

## Abstract

**OBJECTIVES:**

To determine the effects of three sessions of a passive stretching exercise protocol on the muscles of elderly female rats.

**METHODS:**

The effects of the stretching exercises on the soleus muscle were analyzed using immunohistochemistry [tissue inhibitors of matrix metalloproteinases (TIMP), the tumor necrosis factor-alpha (TNF-α), and the gene expression levels using real-time PCR of the transforming growth factor-beta 1 (TGF-β1), collagen type 1 (COL1), and collagen type 3 (COL3)]. Fifteen 26-month-old female Wistar rats were randomly divided into two groups, namely, Stretching (SG, n=8) and Control (CG, n=7). The passive mechanical stretching protocol consisted of a set of 4 1-minute repetitions, with 30 seconds between each repetition (total treatment of 4 minutes), three times a week for 1 week.

**RESULTS:**

Immunohistochemical analysis revealed an increase of 71.4% in the TNF-α (*p*=0.04) gene expression levels for the SG and a 58% decrease in the TGF-β1 gene expression levels (*p*=0.005) in the SG compared to that in the CG. No significant differences were observed between the groups for the immunostaining of TIMP-1 or the gene expression levels of COL1 and COL3.

**CONCLUSION:**

Three sessions of static stretching reduced the gene expression level of TGF-β1, which, owing to its anti-fibrotic role, might contribute to the remodeling of the intramuscular connective tissue of the aging muscle. In addition, immunostaining revealed that TNF-α levels increased in the aging muscle tissue in response to stretching, indicating its effect on stimulating extracellular matrix degradation. These outcomes have important clinical implications in reinforcing the use of stretching exercises in the elderly, considering that the aging muscle presents an infiltration of connective tissue.

## INTRODUCTION

Stretching exercises are considered an important intervention to prevent the loss of mobility, which is related to falls, and should, therefore, be included in exercise programs for the elderly (1). It has been shown that a single stretching session improved the balance and gait pattern in healthy older women (2). Furthermore, when muscle-stretching exercises were performed for more than 8 weeks, 2-3 times a week, it increased the range of motion (ROM) and functional capacity in community and institutionalized older women (3).

Experimental animal models have been used to investigate the effects of stretching exercises on muscle cells and to analyze the mechanisms involved in the regulation of trophism (4,5). Stretching exercises performed on young rats prevent the deposition of collagen 1 and 3 in the connective tissue and increase the cross-sectional area of the muscle fibers (6).

A single stretching session (10 repetitions of 1 min each) in young rats, increased the mRNA myogenic differentiation factor D (myo-D), myostatin, and atrogin-1 levels in their soleus muscles. Also, repeated daily stretching sessions (two, three, and seven sessions) over 1 week increased the myostatin and atrogin-1 gene expression levels (7). However, the effects induced by stretching in aging muscle are still poorly understood.

Static stretching carried out in four 30-second bouts, 5 times a week for 2 weeks, prevented the increase in type I collagen in the injured gastrocnemius muscle of young male rats (8). Furthermore, another study showed that seven intermittent stretching sessions, performed once a day, each with ten 60-second repetitions, increased the matrix metalloproteinase-9 (MMP-9), transforming growth factor-beta 1 (TGF-β1), and myostatin gene-expression levels and the proliferation of connective tissues. Nonetheless, this stretching protocol was not sufficient to inhibit muscle-fiber atrophy or induce sarcomerogenesis of the denervated rat soleus muscle (9). Despite previous reports demonstrating the effects of stretching exercises on the regulation of trophism and connective tissue in the muscles of younger individuals, the stretching-induced molecular mechanisms in aging muscles still need to be understood.

The composition and structure of skeletal muscle changes with age. Fat, connective tissue infiltration, and decline in muscle mass, muscle-fiber cross-sectional area (MFCSA), and myofilament elasticity can be observed (10).

Apart from the infiltration of connective tissue and decline in muscle mass, the chronic low-grade inflammatory state present in the elderly, which is characterized by increased concentrations of proinflammatory cytokines, is associated with skeletal muscle wasting, loss in strength, and functional impairment (11). The tumor necrosis factor-alpha (TNF-α) is a potent stimulator of the muscle RING-finger protein-1 (MuRF1), which mediates sarcomeric breakdown and the inhibition of protein synthesis, suggesting intriguing relationships among altered TGF-β signaling, fibrosis, and muscle aging (12). The cytokine TGF-β signaling pathway is constitutively active in aging myogenic progenitors. A characteristic of cells with an activated TGF-β phenotype is an overexpression of the connective tissue growth factor (CTGF) and subsequent fibrosis (13). The release of TGF-β leads to an increase in extracellular matrix (ECM) deposition and results in fibrosis, but the inhibition of metalloproteinases by tissue inhibitors of matrix metalloproteinases (TIMPs) restricts TGF-β activation, thereby decreasing ECM deposition. It has been suggested that, depending on the MMP involved, increased TIMP levels could also result in ECM accumulation (or fibrosis), whereas low levels of TIMPs lead to enhanced matrix proteolysis (14).

There is still a poor understanding of the roles of the cytokines, TNF-α and TGF-β, in non-contractile and myofiber adaptation in response to acute stretching exercises in aging muscle, as well as that of TIMPs in regulating ECM turnover after acute stretching exercises in the aging muscle. Hence, the cellular and molecular mechanisms involved in the response of aging skeletal muscle to stretching, with respect to connective tissue and muscle mass, remain unclear.

Thus, the objective of this study was to investigate the effects of three sessions of a passive stretching exercise protocol on the soleus muscle using immunohistochemistry, by analyzing the staining patterns of TIMPs and TNF-α, and the gene expression levels of the TGF-β1, collagen type 1 (COL1), and collagen type 3 (COL3).

## MATERIAL AND METHODS

In this study, we analyzed the effects on the soleus muscle of three sessions of a passive stretching exercise protocol, carried out over a week. The TIMP and TNF-α were examined using immunohistochemistry, in addition to the gene expression levels of TGF-β1a, Collagen type 1 alpha 1a (COL1A1a), and Collagen type 3 alpha 1a (COL3A1a).

### Animal care and experimental design

Female Wistar rats were used in accordance with the International Ethics Standard for animal experiments. The study was approved by the local Ethics Committee on animal use (protocol n° 732/2012).

The rats were randomly allocated into 2 groups, namely, the Control (CG, n=7) and Stretching (SG, n=8) groups, with the latter being chosen to receive the stretching exercise protocol. The CG was also anesthetized and positioned on the stretching apparatus to be subjected to similar handling and recovery to that of the SG. All rats were subjected to euthanasia after the one-week experimentation period.

After dissection, the soleus muscle was weighed and divided longitudinally into two equal parts for immunohistochemical analysis and extraction of the total RNA according to established methods (15,20). Immunohistochemical analyses were performed as previously described (15).

The primary antibodies specific for the immunohistochemical reaction used were as follows: TNF-α: human monoclonal TNF-α (1:50 dilution) (MA-091-5, Imuny Biotechnology, Campinas, SP, BR) and mouse monoclonal TIMP-1 (1:100 dilution) (AB1827, Abcam, Cambridge, MA, USA). A tissue known to express the antigen of interest served as a positive control, whereas experiments performed by omitting the primary antibody constituted the negative control.

### RNA isolation and analysis

The RNA was extracted and real-time PCR was carried out according to established methods (7). The primer sequences used were from the following genes: COL1A1a (Forward: ATCAGCCCAAACCCCAAGGAGA; Reverse: CGCAGGAAGGTCAGCTGGATAG), COL3A1a (Forward: TGATGGGATCCAATGAGGGAGA; Reverse: GAGTCTCATGGCCTTGCGTGTTT); TGF-β1a (Forward: CCCCTGGAAAGGGCTCAACAC; Reverse: TCCAACCCAGGTCCTTCCTAAAGTC) and GAPDHa (Forward: CCATTCTTCCACCTTTGATGCT; Reverse: TGTTGCTGTAGCCATATTCATTGT). The GAPDH gene was used as an internal control and its expression did not change under any of the experimental conditions.

The data were analyzed using the comparative cycle threshold (Ct) method, and the delta-delta Ct method (ΔΔCt) was used for the analysis of gene expression using RT-PCR. Initially, the ΔCt of each sample was calculated by subtracting the Ct value of the control gene GAPDH from the Ct values of the target genes (TGF-β1a, COL1A1a, and COL3A1a). To calculate the ΔΔCt, the mean ΔCt of the group was subtracted from the ΔCt value of each sample; to obtain the value of the arbitrary unit (AU), the power of the ΔΔCt value of each sample was calculated. The ΔΔCt method was applied to the averaged Ct values obtained to quantify the relative changes in the COL1A1a, COL3A1a, and TGF-β1a gene expression levels.

### Statistical analysis

The Levene and Shapiro-Wilk tests were used to test for homogeneity and normality, respectively. For parametric data, the comparisons between groups were made using the analysis of variance (one-way ANOVA) and *post hoc* Tukey test. For non-parametric data, the Kruskal-Wallis test was used. The initial and final body weights were compared within the group using the Student’s t-test and the level of significance was set at 5%. All results are reported as the mean±standard deviation, and the statistical analyses were carried out using SPSS 20.0.

## RESULTS

### Immunohistochemistry for TNF-α, TGF-β, COL1, COL3, and TIMP-1

**Figure 1:**
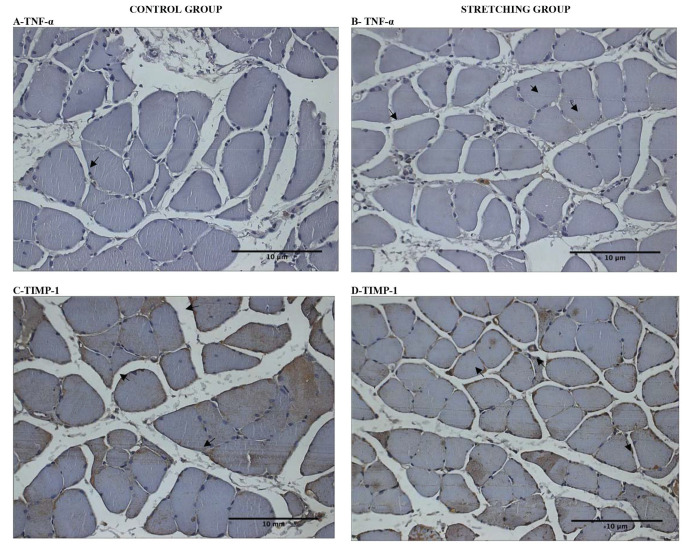
Photomicrographs (x400) of the soleus muscle cross-sections immunostained for TNF-α and TIMP-1. Immunostains are indicated by arrows (→). Scale bar 10 µm (—).

After acute stretching, immunostaining showed an increase in the level of TNF-α (*p*=0.04, Kruskal-Wallis). No difference was detected between the SG and the CG (*p*=0.89, Kruskal-Wallis) for the TIMP-1 immunostaining per soleus muscle fiber area. All data are summarized in Table 1 and the representative samples indicating the percentage of TNF-α and TIMP-1 immunostained per soleus muscle fiber area are presented in Figure 1.

### Changes in the TGF-β1, COL1, and COL3 gene expression levels

After 1 week of muscle-stretching exercises, there was a decrease in the TGF-β1 gene expression level in the soleus muscle of SG rats compared to that in the CG (*p*=0.005, one-way ANOVA). No differences were found in the COL1 (*p*=0.06 one-way ANOVA) and COL3 (*p*=0.17 one-way ANOVA) gene expression levels in the soleus muscle when comparing SG to CG. All data are summarized in Table 2.

## DISCUSSION

This study demonstrated that three sessions of passive stretching exercise of the soleus muscle reduced the gene expression level of the growth factor TGF-β1, an essential mediator of mechanically induced collagen synthesis. Taking into consideration previous results published by our research group, a reduction in the percentage of connective tissue components such as COL1 and in the gene expression level of the growth factor TGF-β1 indicated an anti-fibrotic effect. Furthermore, three sessions of muscle stretching increased the TNF-α levels, as determined using immunostaining.

Immunohistochemistry data showed a higher percentage of TNF-α immunostaining per muscle fiber in the SG than in the CG, which confirms our earlier findings. An increase in TNF-α in the soleus muscle may have contributed to the reduction in muscle trophism, verified after three sessions of stretching exercises (15). This mechanism could be explained by the TNF-α catabolic effect associated with an increased ubiquitin gene expression and protein ubiquitination (21). It has been shown that TNF-α promotes the activation of the nuclear factor κB (NFκB) in skeletal muscle cells. NFκB is a transcription factor that alters gene expressions and causes proteolysis. *In vitro* and *in vivo* data indicate that TNF-α promotes an increase in the gene expression of atrogin-1, leading to the catabolism of the muscle proteins. This is a result of the activation of the ubiquitin/proteasome pathway in the muscle fibers and is believed to be mediated via the p38 MAPK signaling pathway (22). Thus, the atrophy observed could have been mediated by TNF-α via atrogin-1 and the activation of the proteasome pathway. The results of this study point toward the idea of the regulatory role of TNF-α in muscle adaptation after stretching exercises.

TGF-β regulates ECM remodeling and might stimulate the fibroblasts that produce ECM proteins. Concerning ECM remodeling, numerous events contribute to the proteolytic degradation of extracellular compounds, in which MMPs play a crucial role and are inhibited by a number of different TIMPs (5). The TIMPs contribute to the accumulation in connective tissue by the inhibition of MMPs, concomitant with the stimulation of TIMPs by a TGF-β-mediated mechanism (23).

Another finding of the study was a reduction in the gene expression levels of TGF-β1 in the SG as compared to that of the CG without a significant difference in TIMP-1 immunostaining. TGF-β1 belongs to a family of multifunctional proteins and plays an important role in age-associated fibrosis and muscle impairment, and is an essential mediator of mechanically induced collagen synthesis (24). Activated TGF-β induces fibroblasts to produce COL1 and suppresses MMPs (25). These findings suggest that the reduction in the TGF-β1 gene expression may act as a link between stretching and the non-responsiveness of TIMP-1 immunostaining, indicating an anti-fibrotic effect.

Previous findings of our research group showed a decrease in COL1 immunostaining found concomitant with a decrease in TGF-β1 immunostaining after three sessions of muscle-stretching exercises. The results of this study concerning the TGF-β1 gene expression in the SG may contribute to elucidate the role of TGF-β1 in COL1 synthesis. Reduced TGF-β1, as an initial event, could explain the expression of collagen.

In addition, TNF-α reduces ECM deposition either to induce the production of stromal collagenases or to inhibit the synthesis of structural components and COL1, the main structural component of connective tissue (26). TNF-α also counteracts the TGF-β stimulation of the COL1 gene expression (26). These mechanisms, all involved in matrix remodeling, are corroborated by the present results, since the increase in TNF-α immunostaining was accompanied by a decrease in the TGF-β gene expression, while the COL1 gene expression did not change. Furthermore, previous findings contribute to an understanding of the roles of TGF-β1 and TNF-α in COL1 synthesis, in which a reduction in the COL1 immunostaining was observed in the SG, confirming the anti-fibrotic effect of stretching exercises (26,27).

The activated TGFβRI receptor stimulates two signaling pathways, namely, the Smad2/3 and the TAK1 MAPK pathways, which mediate many of the intracellular actions of TGF-β, including the synthesis of the ECM proteins (28). Thus, both previous (15) and current results suggest that the mechanical static passive stretching of the aging muscle decreased the TGF-β1 in the pre- and post-transcriptional levels, which may have contributed to the decrease in COL1 synthesis mediated by TNF-α.

The another study quantified the age‐related alterations in the skeletal muscle ECM and demonstrated that aging was associated with a pathogenic ECM architecture and increased muscle stiffness (29). Another investigation observed an increase in connective tissue and decreases in motor recruitment and contraction velocity of the extensor digitorum longus muscle in elderly rats compared to those in young rats. The research group also carried out immunohistochemical analyses of the COL1 in the plantar muscle and showed that the samples in the elderly group had a significantly higher COL1 level than that in the adult and middle-aged groups. These data indicate that muscle stiffness may also be greater in aging plantar muscles owing to the increased COL1 level in the connective tissue, and further suggest that this affects the functional aspects of the muscle (30). It has been reported that the relative proportion of COL1 increases with age in animals while that of COL3 decreases (31).

In this study, no changes were observed in the gene expression of COL1 and COL3, but another results showed a decrease in the percentage of COL1 immunostaining and greater COL3 level in the SG as compared to that in the CG.

Using ligament fibroblasts, the previous study investigated time-dependent changes in the distribution and gene expression of collagen after the application of 6% cyclic stretching (15). The total RNA of COL1A1, type I collagen alpha 2 (COL1A2), and COL3A1 were extracted from both CG and SG during stretching and at 0, 2, 6, 12, and 18 hours after completing the stretching protocol. Two hours after the cessation of cyclic stretching, the expression levels of COL1A2 and COL3 in the SG were 40% and 71% higher than the respective expression levels in the CG. Six hours after stretching, the expression levels of COL1A1, COL1A2, and COL3 in the SG were 58%, 67%, and 131% higher than in the CG, respectively. Twelve hours after cyclic stretching, the expression of COL1 and COL3 was still upregulated in response to cyclic stretching, but 18 hours after completing stretching, no upregulation in gene expression was observed (32).

Despite the above results having been observed in the ligament fibroblasts, they can, in part, explain the findings of the present study, since no changes were observed in the gene expression levels of COL1 and COL3 in the soleus muscle 24 hours after the last session of the stretching protocol. We believe that the protein levels suggest that the gene expression levels after stretching were time-dependent responses. Although the time point at which the gene expression was measured was relevant, the gene expression was better detected at an early time point after the last session, whereas the protein expression was detected later. This could explain why no change was observed for collagen expression. In the present study, the gene expression levels were only measured after 24 hours and an increase in collagen expression may well have been present at earlier stages. For better results, we believe that the gene expression levels should be determined at an early time point and a western blot analysis can provide information regarding their specificity.

Collectively, all findings indicated that the three sessions of stretching alleviated the TGF-β1 gene expression level concomitantly with a response at the transcriptional level, promoting COL1 and COL3 turnover responses at the transcriptional level. However, the stretching-induced molecular mechanisms involved in connective tissue homeostasis still need to be better understood.

It is plausible that various limitations may have influenced the results obtained. The present study showed the following limitations: 1) the elderly female rats were not compared to younger ones; 2) no functional analysis such as the range of motion was carried out; 3) a biomechanical analysis was not used; 4) no stiffness analysis was carried out to verify passive muscle tension; and 5) no histopathological analysis to determine muscle damage was carried out. Thus, further studies are required to investigate the functional outcomes related to muscle extensibility and the chronic effects of stretching exercises on the aging muscle.

**Figure 2:**
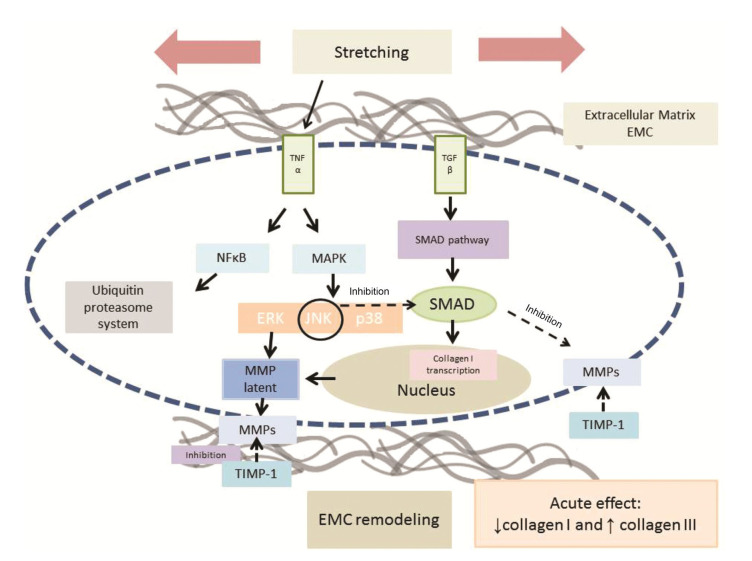
Probable signaling pathways involved in the acute response to skeletal muscle stretching exercises. Stretching stimulus increases TNF-α immunostaining in soleus muscle, and TNF-α might activate the MAPK and NFκB pathways. The MAPK pathway can inhibit SMAD phosphorylation via TGF-β, inhibiting collagen type I immunostaining. Furthermore, TNF-α can inhibit the TGF-β receptor and hence, the TGF-β/SMAD pathway. In addition, MAPK may activate the metalloproteinases which are regulated by their inhibitors (TIMPs). TNF-α can also signal the NFκB pathways that might activate proteolysis through the ubiquitin-proteasome system. The blue dashed line represents sarcolemma. Gray lines represent extracellular matrix. Arrows: activation. Dashed arrows: inhibition. TNF-α: tumor necrosis factor-alpha. TGF-β: transforming growth factor-beta. NFkb: nuclear factor kappa B. MAPK: mitogen-activated protein kinase. SMAD: intracellular proteins that transduce extracellular signals from transforming growth factor beta ligands to the nucleus where they activate downstream gene transcription. ERK: extracellular signal-regulated kinases. JNK: Jun kinase. p38: p38 mitogen-activated protein kinase. MMP: matrix metalloproteinases. TIMP: Tissue inhibitors of metalloproteinases. EMC: extracellular matrix.

Our results suggest that the growth factor, TGF-β1, is an important target related to ECM remodeling and skeletal muscle homeostasis and can be regulated by stretching exercises. Therefore, the findings of both earlier and current studies support the statement that aging muscle adapts to acute stretching exercises via remodeling of the ECM by inhibiting the COL1 content and increasing the percentage of COL3 immunostaining per muscle fiber area, which are mediated by TNF-α and TGF-β1, as summarized in Figure 2.

## CONCLUSIONS

Three sessions of static stretching reduced the gene expression of TGF-β1, which might have contributed, owing to its anti-fibrotic role, to remodeling the intramuscular connective tissue of the aging muscle. In addition, immunostaining revealed that TNF-α levels increased in aging muscular tissue in response to stretching, indicating its effect in stimulating ECM degradation. These outcomes have important clinical implications to reinforce the prescription of stretching exercises for the elderly, considering that the aging muscle presents an infiltration of connective tissue.

## AUTHOR CONTRIBUTIONS

Martins HR contributed in carried out the experiment, analyzed the data and wrote the manuscript. Zotz TG contributed in wrote and supervised the project, helped perform the experiment, analyze the data and write the manuscript. Messa SP contributed in supervised the project, prepared the samples and performed molecular analysis. Capriglione LG contributed in helped carry out animal experiments and prepare the samples. Zotz R contributed in helped care for the animals and prepare the samples. Noronha L contributed in helped supervise the project and assisted in sample preparation and morphological analyses. Azevedo ML contributed in assisted in sample preparation for morphological analyses. Gomes AR contributed in designed and supervised the project, helped perform experiments, analyze the data, and revise the manuscript.

## Figures and Tables

**Table 1:** The acute effects of stretching on the immunostaining of the soleus cross-sections.

Variable	Control Group (n=7)	Stretching Group (n=8)
Percentage of immunostaining per soleus muscle fiber area		
TNF-α Antibody (M±SD)	0.07±0.08	0.12±0.11*
TIMP-1 Antibody (M±SD)	2.72±3.23	2.95±3.10

Data are shown as the mean (M)±standard deviation (SD)

* *p*=0.04 (Kruskal-Wallis) compared with the control group.

**Table 2:** Acute effects of stretching on gene expression levels.

Variable	Control Group (n=7)	Stretching Group (n=8)
Gene		
TGF-β1a (AU)	1.00±0.07	0.58±0.10*
COL1A1a(AU)	1.15±0.61	2.96±0.96
COL3A1a(AU)	1.03±0.30	0.66±0.19

Data presented as the mean±SD

* *p*<0.05, ANOVA compared with the control group. AU: Arbitrary Unit.
